# Design and production of 3D printed bolus for electron radiation therapy

**DOI:** 10.1120/jacmp.v15i4.4831

**Published:** 2014-07-08

**Authors:** Shiqin Su, Kathryn Moran, James L. Robar

**Affiliations:** ^1^ Department of Physics and Atmospheric Science Dalhousie University Halifax NS; ^2^ Queen Elizabeth II Health Sciences Centre Nova Scotia Cancer Centre Halifax NS; ^3^ Department of Radiation Oncology Dalhousie University Halifax NS Canada

**Keywords:** modulated electron radiation therapy, bolus, 3D printing

## Abstract

This is a proof‐of‐concept study demonstrating the capacity for modulated electron radiation therapy (MERT) dose distributions using 3D printed bolus. Previous reports have involved bolus design using an electron pencil beam model and fabrication using a milling machine. In this study, an in‐house algorithm is presented that optimizes the dose distribution with regard to dose coverage, conformity, and homogeneity within the planning target volume (PTV). The algorithm takes advantage of a commercial electron Monte Carlo dose calculation and uses the calculated result as input. Distances along ray lines from the distal side of 90% isodose line to distal surface of the PTV are used to estimate the bolus thickness. Inhomogeneities within the calculation volume are accounted for using the coefficient of equivalent thickness method. Several regional modulation operators are applied to improve the dose coverage and uniformity. The process is iterated (usually twice) until an acceptable MERT plan is realized, and the final bolus is printed using solid polylactic acid. The method is evaluated with regular geometric phantoms, anthropomorphic phantoms, and a clinical rhabdomyosarcoma pediatric case. In all cases the dose conformity are improved compared to that with uniform bolus. For geometric phantoms with air or bone inhomogeneities, the dose homogeneity is markedly improved. The actual printed boluses conform well to the surface of complex anthropomorphic phantoms. The correspondence of the dose distribution between the calculated synthetic bolus and the actual manufactured bolus is shown. For the rhabdomyosarcoma patient, the MERT plan yields a reduction of mean dose by 38.2% in left kidney relative to uniform bolus. MERT using 3D printed bolus appears to be a practical, low‐cost approach to generating optimized bolus for electron therapy. The method is effective in improving conformity of the prescription isodose surface and in sparing immediately adjacent normal tissues.

PACS number: 81.40.Wx

## I. INTRODUCTION

Electron beams provide distinct advantages in therapy of superficial tumors and offer the capacity to spare normal tissues distal to the target volume due to the rapid falloff of the depth dose. Conventionally, the treatment planning variables available to the planner include energy, aperture shape, and bolus thickness. Judicious selection of these parameters produces a plan that provides dose coverage at the surface, as well as at the deep aspect of the PTV, by a high isodose (typically 80%–90% of the maximum dose). However, the dose conformity at the distal surface of the target volume is often poor since the planning and delivery process does not modulate the electron fluence or energy. The consequence is that electron therapy often delivers an unnecessarily high dose to immediately underlying critical structures and tissues. In addition, other than specifying the covering isodose surface as part of the prescription, the dose homogeneity in the target volume is not explicitly controlled during the planning process.

Improved plan quality can be achieved using modulated electron radiation therapy (MERT).^1^, ^2^, ^3^, ^4^, ^5^, ^6^, ^7^, ^8^, ^9^, ^10^, ^11^, ^12^, ^13^, ^14^, ^15^, ^16^, ^17^, ^18^, ^19^, ^20^, ^21^ MERT can be accomplished by sophisticated techniques based on modulation of multiple electron energies and electron beam weights or intensities, which is further classified into two categories: segmented‐field electron conformal therapy (ECT) and intensity‐modulated electron therapy (IMET). Segmented‐field ECT uses multiple electron treatment fields, each with its specific energy and beam weight, to achieve dose uniformity in the PTV.^(2)^ IMET is similar, in concept, to inverse treatment planning (or optimization) of IMRT and uses multiple, optimally‐weighted beamlets to deliver dose conformal to the PTV.^(3)^ Beamlets are shaped by a conventional photon multileaf collimator (pMLC),^4^, ^5^, ^6^, ^7^, ^8^ specific add‐on electron multileaf collimator (eMLC),^9^, ^10^, ^11^, ^12^, ^13^ or few‐leaf electron collimator (FLEC).^14^, ^15^ Though the MERT techniques show promising results in terms of PTV coverage and normal tissue sparing, they are complex in clinical implementation and dose delivery, thus compromising the efficiency of treatment.

An alternative approach involves a bolus with modulated thickness. For MERT using bolus, bolus thickness is optimized by considering the shape of the PTV and the range of electron beam. As a result, a customized prescription isodose surface can be produced within the patient. An effective bolus design algorithm based on an electron pencil beam model was presented by Low et al.^(16)^ Using this algorithm, excellent dose conformity was shown for several cases for bolus produced by a milling machine.^17^, ^18^ However, since the pencil beam calculation is limited in the presence of inhomogeneities or highly irregular patient surfaces, the resulting dose distributions may lack dosimetric accuracy.

The purpose of this study is to explore whether conformal dose distributions can be achieved through modulating the thickness of PLA bolus produced by a 3D printer. In addition to potential improvements in dosimetric quality, the approach may offer practical advantages because i) both the PLA material and the 3D printer involve low capital and consumable cost, ii) the bolus can be generated accurately without the requirement for a session involving both the patient and multiple staff members, and iii) the bolus can be regenerated, if required, at any point during the course of therapy.

In this work we present an in‐house algorithm for bolus design, starting with an initial dose distribution for uniform bolus calculated by electron Monte Carlo (eMC). We examine the efficacy of the approach for regular, geometric phantoms, as well as realistic patient cases. Finally, we assess accuracy with regard to the correspondence of the dose distributions based on the idealized calculated bolus and that resulting from the actual manufactured bolus.

## II. MATERIALS AND METHODS

### A. Bolus design workflow

**Figure 1:**
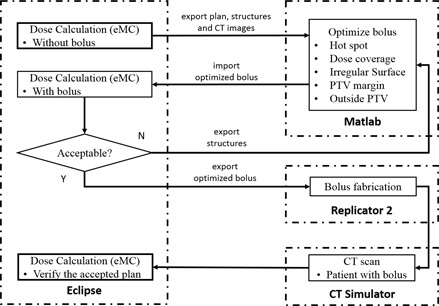
Bolus design workflow.

The bolus design workflow (Fig. 1) starts with creating a conventional electron plan in the planning system (Eclipse version 10.0 Varian Medical Systems, Palo Alto, CA), where the energy and aperture are specified such that the distal part of 90% isodose will cover all portions of the distal PTV. In this initial plan, a 1.0 cm thick bolus is added to define the surface that will be occupied by the bolus; however, the plan is calculated without taking the bolus into account. The dose distribution is calculated using the electron Monte Carlo (eMC) algorithm (Varian Medical Systems), with 0.1 cm grid size, accuracy of 2%, and medium smoothing level. All DICOM objects (images, structures, dose, and plan) are then exported to the bolus design algorithm. This algorithm, implemented in MATLAB (MathWorks, Natick, MA), optimizes the bolus design for the same energy, field size, and source‐to‐surface distance (SSD). The new bolus object is then exported to Eclipse for accurate eMC calculation. If required, this cycle is iterated until an acceptable design is realized. The final bolus is fabricated using a 3D printer (Replicator 2, MakerBot Industries, LLC, Brooklyn, NY) and imaged in place on the patient or phantom using CT (LightSpeed 16, GE Healthcare Ltd., Waukesha, WI) for final dose calculation.

### B. Calculation of bolus design

**Figure 2:**
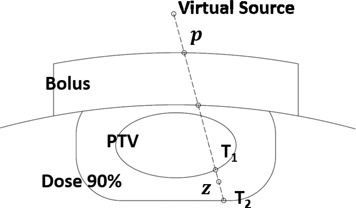
Real distance $T_{1}T_{2}$ along each ray line.

Bolus design (Fig. 2) is calculated on a grid containing the isocenter and perpendicular to central axis. Bolus thickness is calculated using a grid size of 2.5 mm as default; however a finer grid can be used for improved precision. Structures exported from Eclipse (i.e., ‘bolus', ‘PTV’, ‘Dose 90%’ and ‘Hot Spot’ (if required)), are segmented into distal (i.e., deeper) and proximal (i.e., shallower) surfaces according to the maximum and minimum lateral coordinates. Ray lines are traced from the virtual source to each point on the grid, and extended to the distal side of PTV and 90% isodose surfaces. For ray lines intersecting the PTV, the distance $z_{\text{real}}=T_{1}T_{2}$ is calculated. Bolus design for ray lines outside of the PTV is addressed by a subsequent operator (see Material & Methods section C.5 below.)

**Figure 3:**
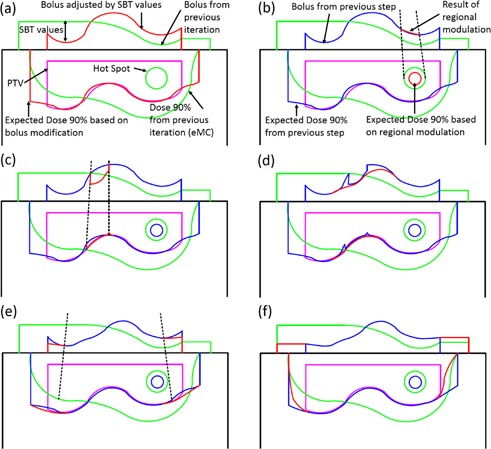
Schematic representation of bolus design algorithm after first iteration. The green lines indicate the previous iteration's bolus and corresponding 90% isodose line which does not yet conform well to the PTV (magenta) in this example. The red lines show the bolus shape modified by the current step (a‐f) (i.e., change in thickness by SBT value or a regional modulation operator), as well as the effect of this change on the dose distribution. For reference, blue lines denote the bolus shape and 90% isodose line from the previous step. Hot spots are indicated as circles. The individual steps are: (a) estimation of the bolus thickness based on SBT values, (b) smoothing for hot spots, (c) smoothing for dose coverage, (d) smoothing for surface irregularity, (e) adjustment at PTV margin and (f) extension outside PTV.

Since patients typically contain tissue inhomogeneities, $z_{\text{real}}$ is converted to an effective distance $z_{\text{eff}}$ using the coefficient of equivalent thickness (CET) method. The effective shift of bolus thickness (SBT) of a certain point p on the grid is given by:

(1) $$SBT_{p}=\frac{1}{CET(Bolus)}\int_{T2}^{T1}CET(z)dz$$

where *CET(z)* is the density at point z relative to that of water. Note that because the initial plan is calculated with no bolus and the requirement is complete coverage of the PTV by the 90% dose surface, all ${\text{SBT}}_{p}$ values will be positive in the first iteration. In subsequent iterations, ${\text{SBT}}_{p}$ values are used to adjust the design of the bolus resulting from the previous iteration (Fig. 3(a)). The density is obtained from the HU to density lookup table in the planning system which, in turn, was obtained during eMC commissioning from a HU calibration phantom (Catphan, the Phantom Laboratory, Salem, NY). The accuracy of the CET method has been discussed in AAPM Report of Task Group 70;^(22)^ the approach is useful for simple geometries, but the accuracy is compromised in the presence of complex inhomogeneities. Each iteration of the algorithm includes calculation by the eMC algorithm such that subsequent modifications are based on an accurate dose distribution.

### C. Regional modulation

**Figure 4:**
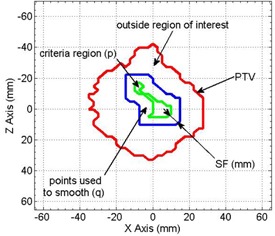
Schematic representation of regions involved in smoothing (e.g., to alleviate a hot spot). The red line shows the projection of the PTV onto the calculation plane. The green line denotes the region of interest satisfying the hot spot criterion and containing points, p, that will be adjusted. Points q between the blue and green lines are included in the smoothing operation, but are not adjusted.

While the calculation of SBT values largely improves conformity of the 90% isodose surface, it does not address secondary effects, such as regional hot or cold spots or the effect of irregular bolus surface. Separate regional modulation operators are developed to address: i) hot spots in the PTV, ii) undercoverage, iii) irregular bolus surface, iv) coverage at the PTV margin, and v) extension of the bolus beyond the PTV. These operators are applied sequentially; however, we reiterate that the dose calculation is performed only by the eMC algorithm in the planning system. Three of the operators (i‐iii) involve regional smoothing. In these cases, the SBT matrix is segmented into regions of interest containing points p where modulation is required, neighboring points q that are used to smooth p, and points outside of the region of interest (Fig. 4). Three smoothing operators are used according to the application:

(2) $$SBT_{p}=\{\begin{array}{l} RM(p,q,SF,Model1)=\frac{0+\sum_{r_{pq}<SF}SBT_{q}\exp(-r_{pq}^{2}/2SF^{2})}{1+\sum_{r_{pq}<SF}\exp(-r_{pq}^{2}/2SF^{2})}\\ RM(p,q,SF,Model2)=\frac{SBT_{p}+\sum_{r_{pq}<SF}SBT_{q}\exp(-r_{pq}^{2}/2SF^{2})}{1+\sum_{r_{pq}<SF}\exp(-r_{pq}^{2}/2SF^{2})} \end{array}$$

where $r_{pq}$ is the distance between p and q, and *SF(mm)* is the smoothing factor, controlling the width of smoothing region and smooth level (i.e., 5, 10, and 20 mm for low, medium, and high).

#### C.1 Smoothing for hot spot

The first modulation operator aims to alleviate the hot spots that exist within the distribution after the previous iteration of eMC dose calculation (Fig. 3(b)). No smoothing is required if maximum dose is less than 110% of the prescription dose; otherwise, the hot spot region is projected to the SBT plane and smoothed. RM(Mode 1) is chosen here since the original SBT value in this criteria region may differ appreciably compared to the surroundings.

#### C.2 Smoothing for dose coverage

Although the calculation of SBT values aims to provide full coverage by the 90% isodose surface, accurate eMC calculation following bolus design may reveal undercoverage in certain regions of the PTV. In these regions, SBT values will be negative (i.e., to decrease bolus thickness). However, testing of the effect of SBT adjustment alone reveals that the bolus thinning must be extended somewhat beyond the region defined by the projection of the under dosed area. Accordingly, negative SBT values in the region of interest are retained, while surrounding values are smoothed (see Fig. 3(c)). RM(Mode 2) is invoked here, which will always increase target coverage since all affected points assume negative values following the operation.

#### C.3 Smoothing for potential irregular surface

Following the previous operations, discontinuities may be present at the boundaries of regions of interest. Surface irregularities are identified by using a gradient threshold criterion equal to two times of the mean value of gradient magnitude, and smoothed using RM(Mode 2) (Fig. 3(d)).

#### C.4 Adjustment at PTV margin

**Figure 5:**
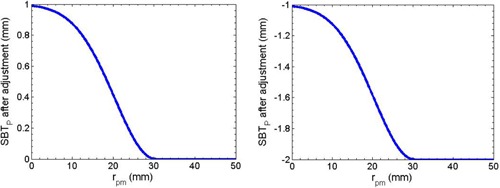
Diagram representation of Eq. (3) using sigma=10 when ${\text{SBT}}_{p}$ before the adjustment is assigned to 1 (left) and −1 (right).

Relative to more central regions, the edge of the PTV receives less scattered radiation dose simply due to collimation by the electron applicator. To remedy underdosing in this region, a region of interest is defined as a 10 mm wide border inside of the projection of the PTV onto the SBT matrix (Fig. 3(e)). A function is applied to reduce bolus thickness according to:

(3) $$SBT_{p}=\{\begin{array}{l} SBT_{p}\times (1-KerfMA(\max(K1-r_{pm},0))),\;if\;SBT_{p}>0\\ SBT_{p}\times (1+KerfMA(\max(K1-r_{pm},0))),\;if\;SBT_{p}<0 \end{array}$$

where values are adjusted along radial lines from the central axis: *m* exists on the inner boundary of the region of interest, *p* exists within the region of interest, $r_{pm}$ is the distance between p and m, $KerfMA(x)=exp(-\frac{x^{2}}{2sigma^{2}})$, and $K1=\sqrt{-2\;\text{ln}(0.01)sigma^{2}}$ (i.e., the distance over which KerfMA(x) increases from 0.01 to 1 (Fig 5)). In practice, we determine that effective values of sigma must be related to beam profile, increasing with both energy and applicator dimension.

In this work and for coding simplicity, an approximation of $sigma=\sqrt{\text{Energy}\times \text{Applicator})}$ is employed.

#### C.5 Shift outside PTV

The area corresponding to all ray lines between the edge of the PTV and a distance 1.0 cm beyond the electron aperture are subject to this operator. In this region, bolus thicknesses are simply extruded:

(4) $$SBT_{p}=SBT_{n}$$

where *n* is the intersection of PTV contour and line from *p* to the projection of central axis (Fig. 3(f)).

### D. Bolus fabrication

The final modulated bolus is converted into a stereolithography (STL) file, which can be provided as input to the 3D printer for fabrication of the bolus using polylactic acid (PLA). A set of parameters selectable within the 3D printer software (Makerware, MakerBot Industries, Brooklyn, NY) are relevant to the printing quality and method and define a print profile. The ‘sparseInfillPattern’ option is set to ‘linear’, which determines the infill pattern. The layer height controls the thickness of deposited PLA in each layer of printing and largely determines printing speed; low, standard, and high quality print profiles correspond to 0.3, 0.2, and 0.1 mm heights, respectively. ‘100% infill’ is used to make the bolus completely solid, otherwise the bolus will be filled a low‐density honeycomb pattern automatically, as controlled by the 3D printing software. The CT value of manufactured bolus is measured to be 160±20 HU, which corresponds to a density of $1.119\pm 0.012\;g/{\text{cm}}^{3}$.

### E. Dosimetric verification

A series of measurements are conducted to acquire the CET value of manufactured bolus by replacing the Solid Water phantom with PLA slabs of varying thickness. CET value of bolus is given by:

(5) $$z_{eff}-z_{real}=[CET(bonus)-1]\times t$$

where *t* is the thickness of bolus slab. The shift $z_{\text{eff}}–z_{\text{real}}$ is determined by matching the location of minimum of $\chi^{2}$ of two PDD curves.^(23)^ Central axis percentage depth dose (PDD) curves are measured with $10\times 10\;{\text{cm}}^{2}$ field size and 100 cm SSD using 6, 9, 12, and 16 MeV electron beam on a Varian 2100EX linear accelerator (Varian Medical, Inc.). Cylindrical chamber (Semiflex 31010, PTW, Freiburg, Germany) and parallel plate chamber (Exradin A11, Standard Imaging, Inc., Middleton, WI) are used to detect the radiation. Three $12\times 12\times 1\;{\text{cm}}^{3}$ PLA slabs are printed. PLA slabs replace the superficial 1 cm, 2 cm, and 3 cm of solid water, for 6 and 9 MeV, and 12 and 16 MeV, respectively. The results of the same measurements are also compared to eMC calculations in the planning system.

### F. Quality assurance

After the bolus fabrication, a CT scan of phantom with bolus added is acquired. The reasons for this step are: i) to verify adequate fitting of the manufactured bolus, and ii) to allow calculation of a final dose distribution (i.e., in case there are differences between the geometry of the calculated and manufactured bolus). The same plan used for bolus optimization is applied to the CT image set with the manufactured bolus. If air gaps exist between the bolus and the patient, the effects of these can be assessed in this final calculation. Here we assess 2D agreement using the gamma evaluation method.^(24)^ The gamma values are calculated in OmniPro I'mRT (IBA Dosimetry, Bartlett, TN) using acceptance criteria of 3% and 5 mm.

## III. RESULTS

### A. CET value of bolus material

**Figure 6:**
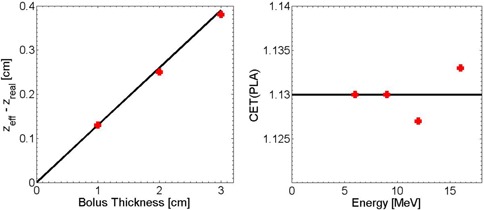
Measured shift $z_{\text{eff}}–z_{\text{real}}$ of PDD curves for a 12 MeV electron beam incident on a Solid Water phantom for 0, 1, 2, and 3 cm thicknesses of solid water replaced by PLA slabs (left). CET value of PLA vs. incident energy of 6, 9, 12, and 16 MeV (right).

**Figure 7:**
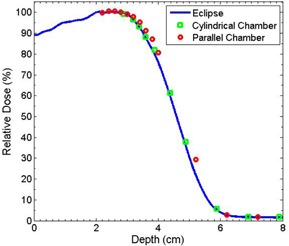
PDD curves for 12 MeV electron beam with 2 cm solid water replaced by PLA slabs.

Figure 6(a) shows the shift $z_{\text{eff}}–z_{\text{real}}$ determined by the differences of CET value between PLA and water according to the measured PDD curves using ionization chamber. No obvious energy dependence is observed (Fig. 6(b)). Therefore, these data are averaged over the four energies to provide a value for the measured CET of 1.13±0.01. The uncertainty mainly results from the minimum thickness of solid water (i.e., 2 mm). This CET value agrees well with the calculated value 1.119 given by the density of the PLA. The measurement using a parallel plate chamber yielded the same result as that for cylindrical chamber, while the eMC simulation gives a prediction of 1.08±0.01. The discrepancy is not expected to produce clinically significant errors in the resulting dose distribution. The coincidence of simulated PDD and measured PDD is shown in Fig. 7.

### B. Wedge target volume with heterogeneity

**Figure 8:**
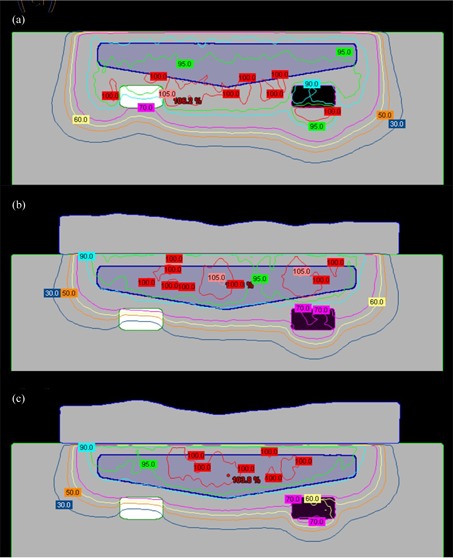
Wedge‐shaped PTV case (a) where a $20\times 20\times 20\;{\text{cm}}^{3}$ water phantom was irradiated by 12 MeV with no bolus; following (b) one and (c) two iterations of bolus optimization.

Several simulations are used to evaluate the algorithm. The first example includes a wedge‐shaped PTV in a water phantom. As shown in Fig. 8(a), two inhomogeneity regions, assigned CT values of bone and air, respectively, are added to the geometry distal to the PTV. A 12 MeV electron beam and $10\times 10\;{\text{cm}}^{2}$ applicator are used. With no bolus, the perturbation of the dose by the inhomogeneities as calculated by eMC is apparent. As shown in Fig. 8(b), after one iteration of bolus optimization, the 90% isodose is almost symmetric about central axis with the 0 HU bolus, though deficiencies in conformity persist particularly proximal to the air inhomogeneity. This likely arises from the simplicity of the scaling provided by the CET method. However, a second iteration of the optimization further improves the conformity of the 90% isodose to the target volume. In practice, even in the presence of tissue inhomogeneities, a conformal dose distribution usually can be achieved in one or two iterations of the algorithm.

**Figure 9:**
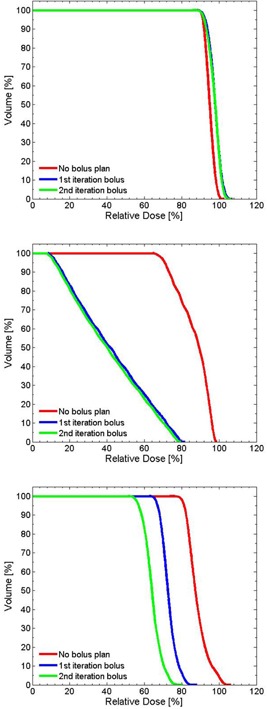
Cumulative DVH for the wedge PTV (top), bone slab (middle), and air cavity (bottom).

In this example, while dose conformity increases, optimization of the bolus surface produces an inadvertent hot spot in the central volume of the PTV (Figs. 8(b) and 8(c)). This is caused by the increase of electron scatter toward the midline by the thicker regions of the optimized bolus. This indicates that the conformity of the 90% isodose line to the target volume and the uniformity of dose distribution may not be achieved simultaneously. Figure 9 demonstrates that, with successive iterations of the algorithm, the coverage of the PTV is not compromised, while the sparing of distal regions (e.g., contoured bone and air volumes in this example) improves. Here, the maximum dose decreases from 98.8% to 79.5% and from 106.3% to 80.4%, for the bone and air inhomogeneities, respectively.

### C. Foot phantom

**Figure 10:**
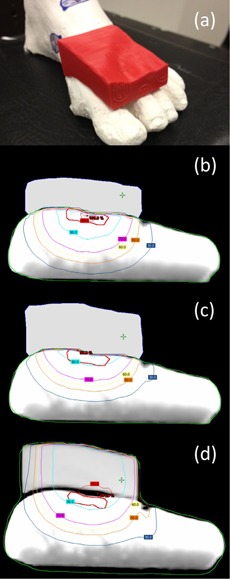
Picture of foot phantom (a) with bolus added on the surface. Isodose plot of (b) conventional plan using 1 cm bolus, (c) MERT plan using optimized bolus, and (d) MERT verification plan using bolus printed by the standard print profiles.

**Figure 11:**
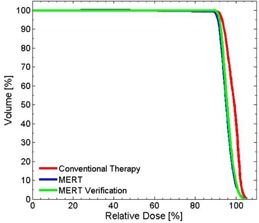
Cumulative DVH for PTV in the foot phantom using planned bolus and fabricated bolus.

For a more realistic geometry, a foot phantom made of plaster was manufactured (Fig. 10(a)). This provides an anatomy that is reasonably complex with regard to the surface curvature, with a simulated PTV at the proximal aspect of the metatarsals. The initial plan uses a 9 MeV electron beam with a $6\times 6\;{\text{cm}}^{2}$ applicator and 1 cm thick uniform bolus to achieve full coverage of the PTV (Fig. 10(b)). The CT value of this bolus is assigned to 160 HU. Figure 10(c) shows the result of one iteration of bolus optimization. DVHs of the conventional (uniform bolus) and MERT plans are shown in Fig. 11. The MERT plan gives 98.4% PTV coverage by the 90% dose level, with a maximum dose of 104.7%. This demonstrates the possibility of good conformity of the prescription dose surface, while maintaining reasonable dose homogeneity.

**Figure 12:**
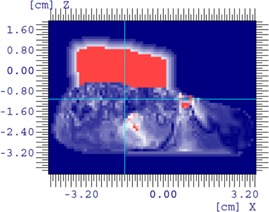
Gamma comparison of MERT using planned bolus and fabricated bolus for foot phantom.

The printed bolus placed on the foot phantom is shown in Fig. 10(a). We observed good agreement between dose distributions produced with the calculated and manufactured boluses (Figs. 10(c) and 10(d)). Between 140 and 200 min are required to print the bolus for low and standard print profiles, respectively. Improved fitting and small air gaps between bolus and phantom surface are observed with the standard profile, and dose distributions are minimally affected. Gamma comparison (3%/5 mm criteria) of dose distributions using planned bolus and fabricated bolus is displayed in Fig. 12, indicating the discrepancies between the plans are acceptable. The high gamma region is attributed to the fact that Eclipse is unable to calculate the dose in a synthetic bolus, since the synthetic bolus is defined as a support structure with a density override.

### D. Head phantom

**Figure 13:**
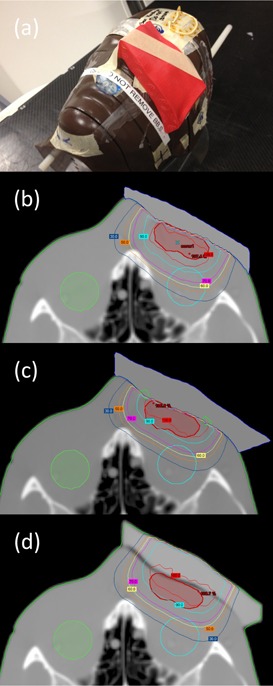
Picture of head phantom (a) with bolus added on the surface. Isodose plot of (b) conventional plan using flat bolus, (c) MERT plan using optimized bolus, and (d) MERT verification plan using bolus printed by the standard print profiles.

**Figure 14:**
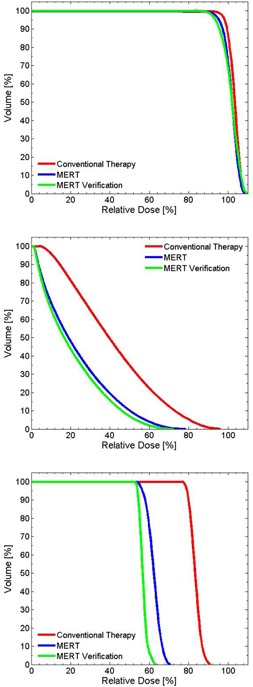
Cumulative DVH for PTV (top), left eye (middle), and left lens (bottom) for head phantom case.

**Figure 15:**
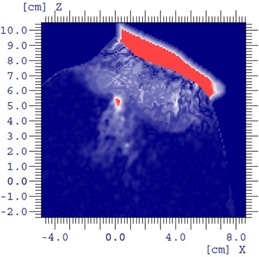
Gamma comparison of MERT using planned bolus and fabricated bolus for head phantom.

A head phantom case is chosen to provide a second assessment of the algorithm in addressing a realistic patient geometry. A simulated PTV is contoured in the head phantom (RANDO Phantoms, the Phantom Laboratory) (Fig. 13(a)). The PTV is irradiated using a 9 MeV electron beam and $10\times 10\;{\text{cm}}^{2}$ applicator incident with a gantry angle of 30°. Note that the ‘eyes' serve as distal organs at risk (despite the somewhat unrealistic position of the eyes due to the embedding of an actual skull within a tissue substitute mold to create the phantom). To represent a ‘standard’ approach, a 160 HU bolus is added to make an approximately flat surface (Fig. 13(b)). The dose distribution of the initial and MERT plans are shown in Figs. 13(b) and 13(c), respectively. For the MERT plan, 90% dose covers 99.7% PTV, and the mean dose for the left eye and left lens decrease from 41.7% to 23.1% and 83.3% to 62.2%, respectively (Fig. 14). The fabricated bolus fitted to the surface is visible in Fig. 13(d). Though a small air gap is observed, the gamma evaluation suggests that the variation caused by the air gap did not compromise agreement of the measured dose distribution (Fig. 15). With regard to the occurrence of air gaps, the two phantom simulations likely represent a worst‐case scenario since both the anthropomorphic phantom and the bolus are composed of hard nonmalleable surfaces.

### E. Rhabdomyosarcoma patient

**Figure 16:**
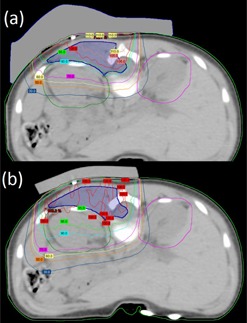
Rhabdomyosarcoma patient (a) using MERT with the algorithm applied for three times; (b) conventional therapy with 1 cm custom bolus.

**Figure 17:**
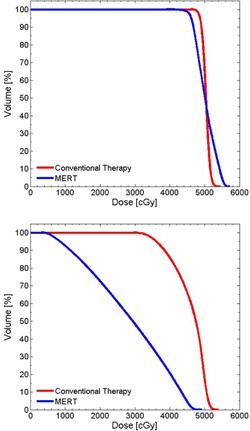
Cumulative DVH for PTV (top) and left kidney (bottom) for rhabdomyosarcoma patient.

Figure 16 shows an example of optimized bolus for a pediatric patient with a rhabdomyosarcoma tumor proximal to the left kidney and overlapping spine. Due to the depth of the PTV, a 16 MeV electron beam was used. 5040 cGy in 28 fractions is prescribed to the 90% isodose covering the PTV. A $10\times 10\;{\text{cm}}^{2}$ applicator and SSD of 105 cm are used. Figure 16(a) shows the result of three iterations of the bolus optimization algorithm plan, while Fig. 16(b) demonstrates the dose distribution that would result from 1 cm thick uniform bolus. The MERT plan allows a reduction of the mean dose of left kidney by 38.2% from 4586.7 to 2834.5 cGy (Fig. 17). While the 90% dose conformity is excellent for the MERT plan, the shape of the bolus generates electron scatter that produces 110% hot spots in the PTV (Fig. 16(a)).

## IV. DISCUSSION

The present work provides a Monte Carlo based algorithm that optimizes bolus design for conformation of the dose distribution to the PTV, addresses dose uniformity, and produces output that can be received by a 3D printer. In comparison to the pencil beam model approach,^(16)^ the eMC calculation used at each iteration will improve the accuracy of dose distribution in terms of the capability of handling the tissue heterogeneity and contour irregularity. The accuracy of eMC for calculation with PLA bolus has been demonstrated. The design algorithm itself does not require dose calculation and thus it would lend itself well to incorporation within a planning system; the output is provided in standard STL format, which is the standard for 3D printers, the cost of which has plummeted in recent years.

The ray line tracing method provides an initial approximation of bolus thickness adjustment at each iteration. However, without further modification it is found that the conformity is generally poor and PTV dose coverage requirements are not met. Accordingly, five regional modulation operators are applied, each solving a specific issue within the same iteration of bolus design. With these adjustments, existed hot spots can be reduced, better target coverage can be acquired, and potential high doses can be avoided. These operators perform on specific regions of interest that are defined by specific criteria; this is in contrast with the approach of modification of the entire target volume less margin (TVLM), as implemented by Low et al.^(16)^

Both idealized and realistic target volumes for electron treatment cases demonstrate the efficacy of the technique. The simulation results of four phantom/patient studies show that the bolus design algorithm can address target volumes with variable shapes, location, and proximity to organs at risk. The main advantage of MERT using optimized bolus over conventional electron therapy is a substantial reduction of the volume of normal tissues being irradiated. This will largely benefit patients who have organs at risk proximal to PTVs.

One concern of this methodology is the potential irregular bolus surface when the distal contour of PTV is extremely variable, which will inevitably result in local hot spots and, consequently, higher maximum dose. The employment of hot spot smoothing operator can alleviate this problem but, in some cases, we found a tradeoff between dose uniformity and conformity.

The 3D printing approach allows accuracy in manufactured bolus at low cost. Compared to the standard process of defining a uniform bolus in the planning system followed by application of generic bolus material, the method allows for a high degree of similarity between planned and actual bolus geometry. No additional information is required beyond the image set, and the demands for both the staff and patient are reduced. In contrast to the manufacture using milling machine,^17^, ^18^ which may be completed outside hospital, a 3D printer can be easily installed in the cancer center, thus likely reducing cost and simplifying logistics of production.

We expect to incorporate this methodology into our own clinical practice for a range of indications. Future work will also address the use of 3D printed bolus to address challenges specific to small field electron therapy.

## V. CONCLUSIONS

In this work, we have investigated a practical approach to MERT employing optimized design and 3D printing of bolus. The method offers practical advantages in that neither patient nor staff needs to be present during the bolus fabrication process. In comparison to manual bolus fabrication (e.g., shaping of synthetic bolus sheets or molding of wax or thermoplastics), the technique allows for optimization of bolus design with regard to dose conformity and homogeneity within the target volume. The algorithm takes advantage of the accuracy of electron Monte Carlo dose calculation to achieve accurate results, while successive iteration of the algorithm allows progressive improvement of dosimetric quality. Phantom and simulated patient studies demonstrate that the technique can achieve excellent dose conformity and acceptable dose homogeneity.

## ACKNOWLEDGMENTS

The authors are grateful for financial support provided by the Dalhousie University Department of Radiation Oncology. The authors would like to thank Cheryl Anderson for eMC planning support, Karen D Adams for being the foot model of our phantom, and David Parsons and Dr. Edwin Sham for assistance in manuscript preparation.
